# Late gadolinium enhancement as an independent predictor of atrial fibrillation in hypertrophic cardiomyopathy (HCM)

**DOI:** 10.1186/1532-429X-11-S1-P204

**Published:** 2009-01-28

**Authors:** Rory O'Hanlon, Agata Grasso, Chiara Bucciarelli-Ducci, Meghana Kulkarni, Susan Clark, Ricardo Wage, Jessica Webb, Michael Roughton, Leena Sulaibeekh, Dana Dawson, Dudley J Pennell, Sanjay K Prasad

**Affiliations:** Royal Brompton Hospital, London, UK

**Keywords:** Atrial Fibrillation, Cardiovascular Magnetic Resonance, Dilate Cardiomyopathy, Gadolinium Late Enhancement, Hypertrophic Cardiomyopathy

## Introduction

Atrial fibrillation (AF) is the most common arrhythmia observed in HCM, developing in approximately 20% of all cases, with an annual incidence of 1–3% per year. The risk of AF developing is 4–6 fold greater in HCM than in the general population. Its' development is associated with an increased risk of systemic thromoboembolism, heart failure and death. Myocardial fibrosis is an important risk factor for the development of atrial fibrillation as demonstrated in post mortem studies. Cardiovascular magnetic resonance (CMR) is the gold standard imaging tool to visualise replacement myocardial fibrosis in vivo. The presence of fibrosis has been shown to be an important predictor of cardiovascular morbidity and mortality in both ischaemic and dilated cardiomyopathies, and more recently an important predictor of arrhythmia as detected by Holter monitoring in HCM.

## Aims

We hypothesised that the presence of myocardial fibrosis is an independent predictor of atrial fibrillation in patients with HCM in a prospective cohort of patients.

## Methods

Between 2001 and 2005, 126 patients with an established diagnosis of HCM were scanned with gadolinium late enhancement. Clinical data, cardiac events, and investigations were collected prospectively to identify outcomes. Relevant data including severity of mitral regurgitation, left atrial indexed volumes, and ejection fraction were noted. The median duration of follow up was 1076 days. CMR volumes, mass, and ejection fraction were analysed using customised software (CMRTools, London). Myocardial fibrosis was assessed using standard late enhancement techniques with gadolinium-DTPA contrast agent (ADD DOSE. The amount of fibrosis was_quantified from sequential short axis slices (base to apex)apex using customised software (MASS, Medis, Leiden). Myocardial epicardial and endocardial contours were delineated. Regions of normal and abnormal myocardium were identified and the total amount of fibrosis was quantified on a per segment basis using the full width half maximum technique. The total amount of fibrosis was expressed as a percentage per segment, as a total in grams, and as a percentage of the total left ventricular mass. A Cox proportional hazard model was applied to correlate the incidence of atrial fibrillation with the presence of myocardial fibrosis.

## Results

Of 126 patients studied, 87 (69%) had detectable myocardial fibrosis. In those with myocardial fibrosis the incidence of atrial fibrillation over the follow up period was 21% vs 5% for those without (p = 0.033). The presence of myocardial fibrosis was associated with an 8 fold increased risk of the development of atrial fibrillation (HR 8.45; CI 1.12–65.53; p = 0.038). Mutivariate analysis demonstrated that fibrosis remained an independent predictor of AF regardless of mitral regurgitant severity, ejection fraction, and LA indexed volumes.

## Conclusion

The presence of LV myocardial fibrosis in HCM is an independent predictor of the development of atrial fibrillation over a median of approximately three years. This may have important implications for risk stratification, decision to anticoagulate, and maintenance of sinus rhythm in this population. A potential mechanism may relate to associated diastolic dysfunction resulting in changes to atrial architecture. It is also possible that LV changes reflect increased atrial fibrosis as part of the disease spectrum but that is more difficult to detect with present techniques.

